# DEVELOPING AGE-FRIENDLY COMMUNITIES: EVIDENCE FROM MULTIPLE CASE STUDIES

**DOI:** 10.1093/geroni/igz038.654

**Published:** 2019-11-08

**Authors:** Patricia A Oh

**Affiliations:** University of Massachusetts, Boston, Boston, Massachusetts, United States

## Abstract

The age-friendly community movement is gaining momentum in the United States. More than 325 communities have joined the AARP Network of Age-Friendly States and Communities or the WHO Global Network of Age-Friendly States and Communities. The purpose of this multiple case study was to explore what influences municipal decision-making about joining a network and how communities mobilize the resources at their disposal to make age-friendly changes after joining. The conceptual model that guided this exploratory study incorporated Kingdon’s policy change model to explore municipal decision-making about joining a formal age-friendly network and resource mobilization theory to explore factors that influence implementation of age-friendly changes after a community joins an age-friendly network. Data was gathered in three in-depth case studies of age-friendly communities in New England-- Brookline, Massachusetts; Newport, Vermont; and Ellsworth Maine. In these three cases, the policy entrepreneur was key to municipal decision-making. Kingdon posits that a single problem definition increases the likelihood that a policy is adopted. However, in these cases, the policy entrepreneur used selective framing to advocate with local organizations and municipal government, a departure from Kingdon’s model. Implications for age-friendly policy adoption will be discussed. Resource mobilization theory posits that implementation of change is dependent on resources and collaborations. Each case had access to different resources, but partnerships were key to moving the work forward (with or without collaborations). The primary resources utilized were relational and ideological. Material resources were less likely to move the work forward. Implications will be discussed.

